# Ameliorating epistemic injustice in practice: Communication strategies in a research project with refugee youth coresearchers

**DOI:** 10.1111/hex.13926

**Published:** 2023-11-29

**Authors:** Elin Inge, Anna Pérez‐Aronsson, Kalid Ibrahim, Reem Aljeshy, Anna Sarkadi, Georgina Warner

**Affiliations:** ^1^ Department of Public Health and Caring Science Child Health and Parenting (CHAP), Uppsala University Uppsala Sweden

**Keywords:** communication, epistemic injustice, observations, patient and public involvement, qualitative research, refugee, youth

## Abstract

**Introduction:**

Many researchers want to include seldom involved groups, such as refugees and youth, in patient and public involvement (PPI), but experience a number of barriers. The PPI research community calls for critical evaluations that are prospective, data‐based and conducted by researchers and public contributors together. In this study, we conducted a longitudinal evaluation of a core activity in all collaborations: communication strategies. The aim was to evaluate the communication strategies adopted throughout a research project with refugee youth coresearchers.

**Methods:**

This article is based on the evaluation of a project where refugee youth were involved in the online adaptation of a group‐based programme for youth with posttraumatic stress. Behavioural observations and field notes collected during the project were analysed with qualitative content analysis and a readability index, and discussed through the lens of epistemic injustice. The article was cowritten by researchers and refugee youth.

**Findings:**

Four manifest categories were identified: Facilitating engagement through coplanning and circumstantial flexibility; Different needs require different channels; It's not just about the channel—facilitation skills matter; Finding a common language opens a communicative flow. In addition, a latent underlying theme reflecting the role of trust was identified: Interactive moments facilitate trust—trust facilitates richer involvement.

**Conclusion:**

At the core of the identified communication strategies were strengthening relationship‐building and actively facilitating involvement. Establishing trusting relationships enabled refugee youth to share input. The communication strategies increased hermeneutical justice by contributing to a common understanding; thus, taking a step towards ameliorating epistemic injustice.

**Patient or Public Contribution:**

This article is a participatory analysis of a PPI process; it was written in collaboration between researchers and refugee youth coauthors, who were all previously involved in the evaluated project.

## 1 BACKGROUND

This article reports on an evaluation of communication strategies in a project with refugee youth coresearchers, utilising patient and public involvement (PPI). PPI in research is here defined as research being carried out ‘with’ or ‘by’ members of the public rather than ‘to’, ‘about’ or ‘for’ them.^1^ In recent years, the PPI research field has focused on evaluating PPI initiatives.^2^ This is dependent on solid evaluations with tools specifically developed for this purpose, to ensure that PPI is conducted in meaningful and ethical ways, in regard to the research, the contributors and the community the contributors represent.

The PPI field has also started to focus on contributor groups who were previously excluded from involvement, such as children, youth and refugees.^3^, ^4^, ^5^, ^6^, ^7^ Youth, in this article, is defined as persons between the ages of 15 and 24 years.^8^ These groups are rarely represented in academia and can contribute with unique input. Researchers are not immune to prejudice and should be careful not to make assumptions about their own neutrality, even when their intentions are honest.^9^ Therefore, involvement with these groups can benefit from being critically evaluated. Involving public contributors in the evaluation can add another level of insight as they bring both the public contributor perspective and their specific lived experience, such as that of being a refugee youth.

### 1.1 Communication in PPI

At the core of each collaboration is communication. In this article, we base our understanding of communication on the Westley and Maclean^10^ conceptual model of communication, which states that the communication process does not begin with the sender, but rather in the environmental factors that influence both the sender and the receiver of a message. Thereby, the model acknowledges the subjectivity in message encoding and decoding, the importance of how a message is communicated as well as the interactive nature of communication, such as how feedback loops affect communication.

In PPI, communication strategies need to consider the underlying epistemic assumptions, communication styles and needs of researchers and public contributors. Reviews on PPI with migrants show the need to respect the contributors' knowledge and priorities as well as communicate in a language all involved understand.^11^, ^12^ This bears similarities to involving youth in research.^13^, ^14^ Although general advice around involving seldom‐heard groups are already covered in PPI guidelines (such as those from INVOLVE), there is a need for practical and specific insights on communication strategies when involving youths and refugees in research.

### 1.2 Epistemic injustice in PPI

This article draws upon Flickers'^15^ work on epistemic injustice, which she describes as ‘a wrong done to someone in their capacity as a knower’. Epistemic injustice occurs when a person's or group's knowledge is systematically undervalued, misrepresented, mistrusted or silenced. Fricker divides the concept into two forms of epistemic injustice. The first form, testimonial injustice, occurs when a person or a group is attributed with less trustworthiness based on identity prejudice. The second, hermeneutical injustice, is related to a person's, a group's or a society's understanding of experiences. When an experience does not fit any existing concept or explanatory model, it can make it difficult to identify and express that a wrong has been done—there is, as Fricker phrases it, a ‘gap in our shared tools for social interpretation’. This is related to the representation of different groups on the arenas where knowledge is developed, such as academia, which some groups are systematically excluded from. Thus, when refugees are excluded from research it leads to academic society having a lack of understanding of refugee experiences and needs.

Fricker exemplifies epistemic injustice using identities such as race and class, while age as a basis for epistemic injustice has been discussed by other scholars. They argue that children experience epistemic injustice within adult‐governed system which are foreign to children's interpretations, such as the health care system.^16^, ^17^, ^18^ These intersecting identities need to be considered when involving refugee youth—although not children—in the adult‐governed academic system.

Efforts to overcome epistemic injustice include developing the skills of a ‘responsible hearer’.^15^ To ameliorate the epistemic injustice faced by children in an adult‐governed system, the adults need to aim to understand the child's interpretative world, as well as reflect on their own identity and position.^17^, ^18^ Burroughs and Tollefsen^17^ suggest that there is widespread epistemic prejudice against children. Adults are in the position to support or fail to support a child as a testifier, and can contribute to epistemic justice by acting as a ‘responsible hearer’.^17^

## 2 AIM

The aim of this study was to perform an evaluation of the communication strategies adopted throughout a research project with refugee youth coresearchers. Through this, we aim to answer the following research questions: Which communication strategies enabled refugee youth involvement in the evaluated project? How did the communication strategies contribute to enabling involvement?

## 3 MATERIALS AND METHODS

**Figure 1:**
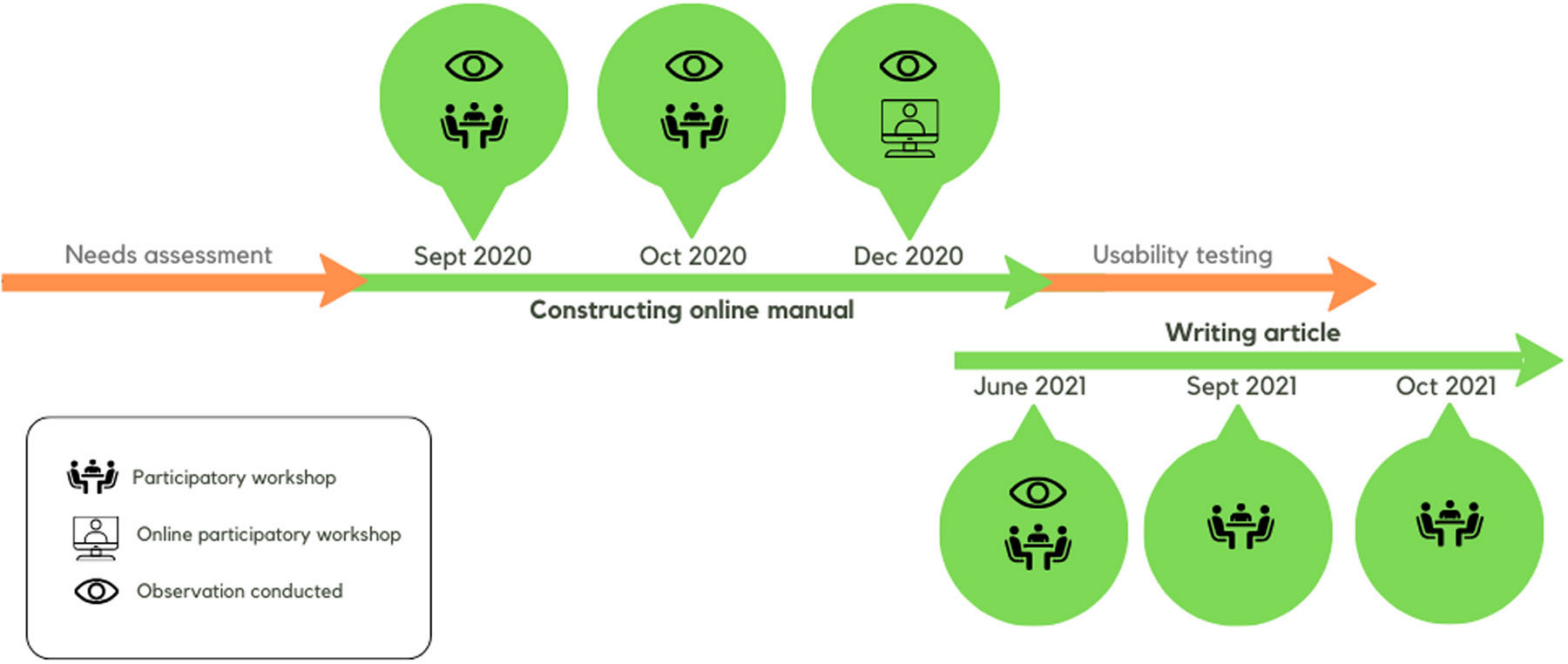
Timeline of project, with the PPI processes covered in this study marked in green. PPI, patient and public involvement.

The setting for the evaluation was a project conducted over 18 months. The project concerned an online adaptation of a manual for support groups for refugee children and youth with symptoms of posttraumatic stress, called Teaching Recovery Techniques (TRT), and coauthoring a research article (Figure 1).^19^, ^20^ The project included a needs assessment through qualitative exploration, using interviews with TRT group leaders and youth who had participated in the groups. This was followed by workshops together with three refugee youth coresearchers, one TRT group leader and two TRT‐trained researchers, to construct a version of the group manual for hosting TRT groups online. The three refugee youth coresearchers were recruited among previous TRT group participants. They were approached by a phone call from one of the researchers, asking if they were interested in working together. Consideration was taken to heterogeneity in individual traits and demographics, such as gender, country of origin and arriving in Sweden unaccompanied or with family. The young coresearchers, one young woman and two young men, were however similar to some extent; they originated from the MENA region, were fluent in Swedish and had attended school in Sweden for some years. The nonresearcher TRT group leader was recruited amongst the local TRT group leaders based on his experiences of having co‐held TRT groups that were moved online due to COVID‐19.

When two of the refugee youth coresearchers were asked to reflect on their involvement in the project, they wrote together: ‘We were about eight people, two of them sat quiet because they wanted to write and hear what we said, and me and the others shared ideas to improve TRT online, it was great fun. Everyone respected each other, everyone listened to each other and most importantly, we all wanted to come and had good energy’.

Usability testing of the manual was performed in two cycles; testing the adapted manual with intervention leaders, and testing the newly developed digital resources with another group of youth. An advisory panel consisting of refugee parents as well as professional and academic experts in the field, was regularly consulted to provide recommendations on the adaptation of the manual. Further details of this project's process and outcomes have been reported elsewhere.^20^

### 3.1 Study design of the PPI evaluation

The study was conducted through a multimethod qualitative approach.^21^ After the project finished, two of the refugee youth coresearchers agreed to cowrite this article; they are hereafter referred to as the refugee youth coauthors. This article focuses on the PPI processes with the refugee youth coresearchers when developing the online manual, including the workshops, and cowriting the previously published article^20^ (Figure 1).

The data consisted of field notes from behavioural observations, researcher notes and documentation and documented communication between team members. The behavioural observations were conducted as part of a wider project, using an observation protocol developed to assess aspects of group dynamics in the context of PPI research meetings.^22^ Two external researchers attended the workshops to conduct passive observations. Both observers were trained TRT group leaders, and had met with both researchers and contributors previously. Four workshops were observed, of which one was online (Figure 1). The first article writing workshop was also observed, after which a decision was taken not to continue observations—with few participants, it was difficult to conduct observations without affecting the workshop. In this study, both field notes assembled under the structured headings of the protocol as well as other notes were used. In addition, the data set included researcher notes taken during, after and between workshops, and documented communications between team members; letters, emails, texts and WhatsApp messages. This data was used to explore the team communication between workshops.

### 3.2 Analysis

An inductive qualitative content analysis, according to Graneheim and Lundman^23^ was used to evaluate communication strategies. The data were sorted chronologically and read repetitively while noting which channel (i.e., mail, WhatsApp etc.) was used for which kind of communication. Relevant meaning units, defined as sentences or paragraphs that relate to a central meaning, were identified and condensed into a description close to the data. These were compared and abstracted into categories focusing on the manifest content, that is, the immediately visible content.^23^ A latent underlying theme was identified, and both the theme and categories were refined during analysis meetings.

All coauthors were involved in the analysis through a process of reflection and discussion in analysis meetings. The two refugee youth coauthors' involvement in the analysis was guided by methods on involving children in analysis.^24^ Together with the first author, they went through the entire data set and decided on analysing parts of the field notes data, from both the manual development process and the article‐writing process in the project (Figure 1), as well as the WhatsApp data. This decision was based on their ability and preferences as well as on team discussions to ensure that they analysed data from different steps in the project. They then participated in three analysis meetings, reflected about how they interpreted the data, discussed categories and gave additional suggestions; for example, the underlying theme was suggested by the refugee youth coauthors.

In addition, all material communicated to the young co‐researchers during the evaluated project was analysed for level of readability using the standard method Swedish Readability Index.^25^

### 3.3 Researcher positionality and reflexivity

A different author team might have reached different conclusions with the same data, as with all qualitative analysis. The academic researchers all work in a research group aiming to improve the mental health and wellbeing of children, youth and parents. They have backgrounds in the health field (Medicine, Nursing, Psychology), are committed members of the participatory research community and have an interest in migration and health. Although none of them are refugees, two have migrated to Sweden and one has a refugee parent. The refugee youth coresearchers have recently graduated from high school and have lived in Sweden for several years. The full author team previously worked together on the evaluated project, giving them an insider lens in the analysis. However, how close they worked to the project varied—A. S. had a senior advisory role, G. W. oversaw the study design, E. I. performed the observations and A. P. A. was responsible for much of the communication and practical work together with refugee youth coauthors K. I. and R. A. Thereby, some of the academic researchers could take a more distant perspective. In the conceptual and analysis phases, these different experiences were identified and discussed with the author team. Analysing and writing together required both academic researchers and refugee youth coauthors to view and negotiate their positions as well as actively acknowledging other team member's positions. It was clear that the insider perspective, predominantly provided by the refugee youth coauthors, provided depth and new directions to the analysis, while the more distant perspective gave clarity and more transferable insights.

## 4 ETHICAL CONSIDERATIONS

Generally, ethical approval is not a requirement for PPI according to Swedish legislation, as public contributors are not research participants. However, the refugee youth co‐researchers were not only involved as public contributors, but in the context of this study they were research participants, along with some of the academic researchers. The observations occurred during workshops about a sensitive topic—an intervention for youth with posttraumatic stress—hence sensitive information risked being recorded in the observation protocol. Therefore, ethical approval from the Swedish Ethical Review Authority was sought and gained both for the evaluated project and for this study.

Additional ethical considerations around involving and working with the contributors were taken. All refugee youth coresearchers involved were initially recruited based on their previous participation in the group programme, which meant that they had a history of trauma and of symptoms of posttraumatic stress. Therefore, we opted for recruiting young coresearchers who were in stable health, both objectively and self‐assessed, at the time of recruitment. When working with the manual in the evaluated project, we were cautious not to expose them to unnecessary reminders, and we limited the discussions to ‘TRT experiences and suggestions’, not ‘trauma experiences’. The research team has experience in involving youth in research, as well as working with children and youth with traumatic experiences. In case any of the contributors would start experiencing posttraumatic stress disorder symptoms as a result of their involvement, there were psychologists and other health professionals in the team, as well as a safety protocol with referral to psychiatric services.

## 5 FINDINGS

The findings are presented as four manifest categories followed by one latent underlying theme (Table 1).

**Table 1:** Study findings.

*Categories*
Facilitating engagement through coplanning and circumstantial flexibility.
Different needs require different channels.
It's not just about the channel—facilitation skills matter.
Finding a common language opens a communicative flow.
*Underlying theme*
Interactive moments facilitate trust—trust facilitates richer involvement.

### 5.1 Facilitating engagement through coplanning and circumstantial flexibility

The planning of the communication strategies was done in collaboration between the researchers and refugee youth coresearchers, both at the onset and continuously throughout the project. The youth were invited to be involved through a phone call, followed by a physical meeting: the first workshop. There, the researchers initiated a discussion on team communication between workshops. The young coresearchers and the service provider stated that using WhatsApp would suit them best and offered to arrange for a group chat for the team. WhatsApp and physical workshops were initially decided to be the main communication channels, but as the project progressed, a need for additional communication channels was identified. For example, when the COVID‐19 pandemic hindered physical meetings, the team agreed to move one workshop online rather than waiting until a physical workshop was possible. In this way, engagement was facilitated by both planning together with the refugee youth coresearchers and allowing for circumstantial flexibility.

Another form of coplanning was the flexibility within workshops. Using workshops was a researcher‐led decision, but the practicalities were adapted to the young coresearchers' preferences. These adaptations regarded time and place as well as researchers helping to solve practicalities, including contact with school if the young coresearchers needed to be absent to join a workshop. Often, the coplanning was outspoken, that is, the team discussed improvements together and acted on them, but the researchers also displayed an awareness of how methods were experienced by the refugee youth coresearchers. For example, the second workshop utilised ‘topic stations’, a method where meeting participants move around the room individually and write down suggestions on different stations. However, the young coresearchers preferred a discussion‐based workshop and the researchers planned the next workshop accordingly. Another example was that the researchers deliberately kept the workshops open for coplanning, to engage the young coresearchers in influencing discussions.

Researcher to refugee youth coresearchers: ‘That is so important, I think we need to talk more about that’. The researcher follows up with the other meeting participants and changes the focus of the meeting to discuss the new suggestion.

(Observation note from workshop)

### 5.2 Different needs require different channels

In the communication channels used, the content and tone varied. The channels complemented each other, as they filled different communicative purposes.

In the WhatsApp chat, much of the content focused on practicalities, such as communicating news in the project, informing about upcoming events or planning meeting times. The researchers also used this channel for more direct project‐related question, for example preparing the refugee youth co‐researchers for an upcoming workshop by sending questions to consider beforehand. In addition, the chat was used to involve the youth in project‐related tasks between workshops, such as asking for their help in finding pictures to use for a relaxation technique, or asking for their advice on the online intervention.

Researcher: Hi! We are talking about online TRT and have a question for you 🤗

You may remember that we talked about recording videos showing some of the exercises, which can be used at home if one wants to practise between meetings. It would be for example the relaxation exercise

We are thinking about who should record the film: should it be a TRT leader like us or X and Y who make the film? Or should it be a young person?

What do you think other young people or children would think would be best?

Youth 1: I think it would be better if a young person did it.

Youth 2: I agree with (Youth 1) it is better if a youth does it

Researcher: Thanks for the quick reply 😊

(WhatsApp data)

The workshops were crucial for the main body of work with the manual and article writing, with more in‐depth project‐related discussions. The observations of the workshops showed that the researchers actively asked the refugee youth coresearchers for input during the discussions as well as on specific ideas. They contributed actively, often spontaneously and sometimes after being probed. Their input consisted of ideas and solutions, responses to researchers and other contributors, and they occasionally challenged research ideas. The researchers responded by summarising the young coresearchers' input to ensure they understood it correctly, asking follow‐up questions, taking notes and feedbacking how they planned to act on the feedback.

Researcher: Writes down ideas, follows up with questions. ‘Is this what you meant?’

(Observation note from workshop)

Phone calls, video‐conference and emails were used to a lesser extent and were used when communication through workshops or WhatsApp was not feasible. For example, phone calls were used when a young coresearcher had missed a workshop and a researcher called to update them and ask for their input. Additionally, it was better to send larger documents and longer texts via email or post instead of WhatsApp. However, that this was less convenient for the refugee youth coresearchers was evident as researchers still needed to use WhatsApp to remind the youths of the emails.

Thereby, the channels filled different communicative needs but all had a role in the overall communication strategy.

It's not just about the channel—facilitation skills matter

Even if the team jointly identified which channels to use, the communicated content was essential. In the researcher notes, how to facilitate engagement and make the material accessible for everyone in the team to be able to contribute with relevant input was considered a challenge. The researchers adopted communication strategies to achieve this, which have been identified in the data.

All material sent out to the refugee youth coresearchers, for example, preparations for workshops or updates on project progress, followed a similar structure. The researchers started the communication by reminding the youth on something they discussed previously, continued with some background information, and then arrived at the current issue. When needed, this also included detailed practical instructions, such as how to prepare for and access an upcoming online workshop. In addition, the researchers always clarified which expectations they had on the young coresearchers, which the youth had specifically asked for.

We have attached a short summary of the suggestions from the professionals. You don't need to read it beforehand, we will tell you more in the meeting. We would like to know what you think about what they said.

(From letter in preparation of workshop)

Another example of a structure was that a mind map was used to summarise workshop discussions back to the participating researchers and refugee youth co‐researchers. The mind map was constructed with short sentences and had colours representing different themes. This was a visual method that everyone was familiar with and that had been previously used by the youth in the school setting.

### 5.3 Finding a common language opens a communicative flow

The language in the material sent out was written in an accessible way. All material shared with the refugee youth co‐researchers during the project was scored between 23 and 37 on the Swedish Readability Index, indicating it was ‘Very Easy to Read’, comparable to children's books, or ‘Easy to Read’, comparable to fiction and popular magazines. During the cowriting process, a researcher made the manuscript more accessible by writing a short Swedish version for the young coresearchers to read together with the English manuscript. The youth stated that this was essential for their involvement in the cowriting process.

Additionally, the researchers often used terms that the refugee youth coresearchers themselves used, contributing to the common language used within the project. One example came from the second workshop, where the topics were based on the summarised discussion from the last workshop, using the same terminology that the young coresearchers and service provider had used. Another example from the cowriting process was when the researchers started with introducing what a scientific article is, and what is required from those listed as authors. The young coresearchers responded with drawing similarities to lab reports they had written in school, which was then used as an example during the cowriting process.

Adapting the languages seemed to fill two connected purposes: making the material accessible and making the refugee youth coresearchers more comfortable. When the researchers used words that the youth were familiar with, they felt comfortable to speak their mind freely without having to be concerned about phrasing or expressing themselves in a correct way.

### Interactive moments facilitate trust—Trust facilitates richer involvement

When evaluating the communication strategies, the commonalities between the strategies that succeeded in facilitating involvement, and those that did not, directed our attention towards a latent, underlying theme. The successful communication strategies were interactive and made the youth feel safe enough to trust the researchers, which in turn made them feel comfortable to speak their mind more freely.

When coplanning the team communication, the refugee youth coresearchers strongly favoured two communication channels: workshops and the WhatsApp group. These channels were highly interactive and allowed for a more personal communication style, with interaction both between researchers and youth as well as between the refugee youth coresearchers. Other channels, such as email or video‐conferences, allowed less interaction, were less appreciated by the team and appeared to have a negative effect on the discussion.

In the workshops, the observations showed that many of the interactions were personal rather than professional, especially in the early collaboration. This included humour, sharing personal experiences during breaks and displaying interest in each other as persons. The interactions were facilitated by the researchers, for example, by arranging to have lunch together, or arranging the seating to be informal and allowing for interactions. The refugee youth coresearchers appeared more comfortable and provided more input when discussing in a team compared to when writing individually, as was the case mentioned above with ‘stations’ for individual contribution at a workshop that the youth preferred changing to interactive discussions for following sessions. The young coresearchers themselves also associated this to being encouraged by receiving direct feedback. This aligns with the observations notes from the workshops—there was plenty of positive feedback observed.

In the WhatsApp group, the tone was informal and relaxed, with short sentences and both researchers and young coresearchers expressing emotions through emojis. Even when the content focused on practicalities, this was often combined with checking in on the youth, for example asking how school is going. In the researcher notes, an explicit aim to maintain contact with the refugee youth coresearchers regularly was identified, even when they had no tasks or direct questions.

Researcher: When is a good time for us to meet? I think you need some time to read before 😃

Youth: Now I have a lot to do at school 😣😣😣😣😣😣😣

So I don't really know when we can meet 😩

😍

Researcher: Ugh yes it can be stressful at the end of the semester!!

(WhatsApp data)

Additionally, how a channel functioned did not seem to depend solely on the channel itself, but also on *who* and *how many* participated in the channel. Throughout the study, two different WhatsApp groups were active, of which one—focused on the cowriting process—was limited to only one researcher and two refugee youth. This smaller group had a more personal tone, which appeared to be positive for relationship‐building and for the young coresearchers to express themselves.

When the researchers expressed understanding for the refugee youth coresearchers, for example showing compassion when they cancelled a meeting due to personal circumstances, it made them feel safe to express research input freely—but also to ask other questions. An example of this, brought up by the refugee youth coauthors during analysis, is that this made them feel comfortable to ask the researchers for advice on summarising their involvement for their resume. A connection between finding a common language and trust was also identified. When researchers communicated in an accessible language and used appropriate communication strategies, the refugee youth coresearchers felt safe to express their thoughts in their own words—which thereby facilitated more interaction and communication.

## 6 DISCUSSION

### 6.1 Discussion of findings

In this study, we evaluated the communication strategies used in a research project with refugee youth coresearchers. We identified some communication strategies, including using coplanning around communication, different channels and facilitation skills, as well as finding a common language. As a theme underlying these strategies, we found that successful communication strategies were interactive and thus contributed to trust, which made refugee youth co‐researchers feel comfortable to speak their mind more freely.

Involving refugee youth in forming the research about them and their experiences is one way to reduce epistemic injustice on a systemic level, through combatting the exclusion of their voices and perspectives in academia. As this is a population experiencing testimonial injustice at group level, the researchers' efforts to involve refugee youth, listen to and value their suggestions, show efforts to act as ‘responsible hearers’ to alleviate epistemic injustice.^15^, ^17^

Our findings suggest that communication strategies within research collaborations can contribute to ameliorating hermeneutical injustice, by bridging the ‘gap in our shared tools for social interpretation’.^15^ This was done by building trust, cocreating inclusive communication strategies and by adapting the form and language of the team communication so that understanding it was not dependent on an academic degree. A core example is allowing the young coresearchers to guide the terminology and explanatory models in the findings, which is related to ‘entering their interpretative world’.^17^, ^18^ The researchers adapted the common language through inviting the young co‐researchers to respond using their own words; the youth did not need to uphold any linguistic standards but could share their thoughts freely. Another example is being flexible to how the refugee youth coresearchers wanted to work in the project, allowing them to contribute in ways that suited them best. These strategies contributed to a mutual understanding and thus increased hermenutical justice.

In the findings, it was clear that the relationships between the researchers and the refugee youth coresearchers was central for the communication strategies to support the epistemic position of the youth. In the underlying theme, this is portrayed as a positive cycle of allowing interaction, including personal rather than professional interaction, to be a central part of the communication. This supported the development of mutual trust which in turn led to richer involvement. Similar insights were previously discussed by Burroughs and Tollefsen,^17^ who stated that relationships is central to the epistemic position of children. This was also seen in youth involvement in mental health research^13^, ^26^ and in involvement with refugees.^11^, ^27^ It is well‐established that conducting PPI in an ethical and meaningful way, takes time and commitment with relationships that might last beyond the project itself.^28^ This requires researchers to step away from their established ways of working with research, learn new skills, such as communicative skills. Forming trusting relationships requires researchers to reflect on their own identity and position, but also to take active steps from reflection to action. Our findings indicate that adopting good communication strategies can be part of building trusting relationships.

The idea of relationships as a basis for all PPI, connects to the need for a flexible approach to the methods used. As Brydon‐Miller et al.^29^ phrases it—researchers working with participatory approaches need to ‘be able to handle a certain degree of chaos, uncertainty and messiness’. PPI activities rarely allow themselves to be fully planned, but are rather dependent on flexibility, knowledge and experience in the team, and on how relationships develop over time.^14^, ^28^ In our findings, flexibility to the team's needs included coplanning team communication, flexibility around meeting times and flexible solutions for involving youth when they missed a meeting. In addition, the team developed their collaboration over time. For example, how the young coresearchers would be involved in cowriting was not decided beforehand, but discussed together with them, taking their skills, availability and wishes into consideration. A practical suggestion for building flexibility into PPI, is to plan for formative process evaluations throughout the project, as suggested by McCabe et al.^13^ and Pavarini et al.^26^ Ideally, a team should decide together on outcomes for an evaluation.

Much of the communication was structured in a specific way, including the researchers communicating which expectations they had on the refugee youth coresearchers. The structure is very similar to communication strategies in health care settings and is likely related to the researchers' medical background. Health care communication tools such as SBAR have been shown to improve team communication in health care settings^30^ but the strategies themselves are generic and useful for most settings. As researchers are trained to communicate in an academic setting, aquiring communication skills for a more general audience along with using an accessible language, appear to be useful in PPI processes with refugee youth.

#### 6.1.1 Advice for refugee youth and researchers on working together

Based on their experiences of involvement as well as working with the findings in this study, the refugee youth coauthors summarised advice for youth and researchers on working together.

It is important that researchers and young coresearchers:

1. Respect each other's time, show flexibility and understand that other things in life sometimes need to be prioritised,
2. Are honest and do what they have committed to, and
3. Stay positive and show that they genuinely want to work together.

Researchers can help through creating a meeting environment where refugee youth feel comfortable to speak up when they have an idea or opinion, as young people may feel uncomfortable speaking in the company of adults.

If they have not met the researchers before, young coresearchers may feel uncomfortable and nervous. Researchers can help through:

1. Giving youth a call before, to get to know them,
2. Planning a social activity together with them for the first meeting,
3. Providing something to eat and drink in long meetings, and
4. Making sure there is more than one young person in each group.

It is easier to talk in the meeting if you have had time to prepare beforehand. Researchers can send a short text about two days before the meeting, giving the youth time to think. Ask the youth through which channel they want to receive the text.

### 6.2 Methodological discussion

In this article, we used observations, field notes and communicated material to evaluate communication strategies in a project involving refugee youth coresearchers. These were analysed with well‐established methods: qualitative content analysis^23^ and a standard readability index.^25^ A core strength of this article is the author team, where both researchers and refugee youth participated. Performing this kind of ‘self‐evaluation’ can be challenging, as already established relationships could potentially hinder voicing negative opinions due to loyalty or fear of risking relationships. On the other hand, established relationships are most often positive for participatory collaborations, as they are the basis of trust.

Observations can make people aware of and change their behaviour. To limit this, the observers placed themselves so that they could see the workshop participants, while those were facing each other. In the online workshop, observers turned their cameras off after introducing themselves. Second, the observers had met the participants before observations, which appeared to increase comfort. Lastly, the observers emphasised that the observations did not aim to evaluate individuals, but rather record the processes.

PPI is at its core a relational activity and not a methodology. Therefore, relationships must come first and evaluations of PPI activities second, which guided our decision to not continue imposing observations on the writing workshops with the refugee youth co‐researchers. With so few meeting participants, external observers risked affecting team relationships, including social interaction, comfort and trust.

The field notes were rich and detailed—a strength in this study. However, collecting more structured field notes is advised. Log books are a well‐established way of tracking involvement processes under structured headings and could reveal more of the underlying mechanisms affecting PPI. An interesting venture would be to encourage PPI representatives to keep logs. However, this time‐consuming exercise needs to be balanced against other project tasks.

## 7 CONCLUSIONS

This study aimed to evaluate the communication strategies used in a research project with refugee youth coresearchers. The identified communication strategies included coplanning team communication, using different channels for different needs, using facilitation skills, and adapting to a common language. When working with refugee youth coresearchers, researchers need to be committed to the PPI process and actively work to build relationships. This includes being flexible to the needs of the team and making the material accessible, which can be achieved through communication strategies. The underlying theme showed that successful communication strategies were interactive and therefore built trust. When trusting relationships were established, the refugee youth coresearchers could share their thoughts, which facilitated richer involvement. These communication strategies increased hermeneutical justice, by contributing to a common understanding; thus, taking a step towards ameliorating epistemic injustice.

## AUTHOR CONTRIBUTIONS

Elin Inge performed observations, took field notes, analysed data and wrote the first draft of the article. Anna Pérez‐Aronsson led the codesign workshops and the cowriting process, took field notes, took part in the analysis and reviewed the article. Kalid Ibrahim and Reem Aljeshy participated in the codesign workshops and the cowriting process, participated in the analysis, wrote the section about ‘Advice for refugee youth and researchers on working together’ and parts of the background, and reviewed the article. Anna Sarkadi took part in the analysis and reviewed the article. Georgina Warner led the planning of the codesign workshops, took part in the analysis and reviewed the article.

## CONFLICT OF INTEREST STATEMENT

The authors declare no conflict of interest.

## ETHICS STATEMENT

Ethical approval from the Swedish Ethical Review Authority was sought and gained (Ref. 2020–03911). The evaluated project, including the codesign process, had also been approved by the Swedish Ethical Review Authority (Ref. 2020‐03126; Ref. 2020‐06693).

## Data Availability

Research data are not shared. First, consent was not sought to share data before data collection, and second, observation notes and WhatsApp conversations with a limited number of participants can make individuals identifiable. The data that support the findings of this study are available from the corresponding author upon reasonable request.
